# Identification of genetic alterations in couples and their products of conceptions from recurrent pregnancy loss in North Indian population

**DOI:** 10.3389/fgene.2023.1155211

**Published:** 2023-05-16

**Authors:** Priyanka Srivastava, Chitra Bamba, Seema Chopra, Minakshi Rohilla, Chakshu Chaudhry, Anupriya Kaur, Inusha Panigrahi, Kausik Mandal

**Affiliations:** ^1^ Genetic Metabolic Unit, Department of Pediatrics, Advanced Pediatric Centre, Post Graduate Institute of Medical Education and Research, Chandigarh, India; ^2^ Department of Obstetrics and Gynaecology, Post Graduate Institute of Medical Education and Research, Chandigarh, India; ^3^ Department of Medical Genetics, Sanjay Gandhi Postgraduate Institute of Medical Sciences (SGPGIMS), Lucknow, India

**Keywords:** recurrent pregnancy loss, next-generation sequencing, chromosomal microarray, copy number variations, chromosomal aberrations, sequence variations, multiplex ligation-dependent probe amplification

## Abstract

**Background:** Recurrent pregnancy loss (RPL) is one of the most common pregnancy-related complications, which can be stressful and emotionally draining for a couple. Genetic alterations, which are responsible for RPL, can be present in either of the three genomes: mother, father, or their fetuses. In addition, environmental factors interacting with these three genomes can affect germline cells. With this aim, the present study was conducted to understand the underlying etiology of RPL using Next-generation sequencing (NGS; couple exome and TRIO exomes) in combination with cytogenetic tests [karyotyping and chromosomal microarray (CMA)].

**Material & Methods:** In present study we recruited 61 couples with RPL (history of ≥ 2 abortions) and 31 products of conceptions (POCs). For all couples karyotyping was done at the time of recruitment, followed by collection of POC samples and parental blood samples. Before processing POC samples for CMA, they were checked for maternal cell contamination (MCC) by QF-PCR. In POC samples with no pathogenic variant, TRIO exome sequencing was done. Further, in case of unavailability of POC sample, couple exome sequencing was done for RPL couples.

**Results:** In six individuals out of 61 couples (5%), abnormality in karyotypes was detected. Among 116 normal karyotypes, there were 11 heteromorphisms (9.5%), for which the couples had to be counselled and reassured. Out of the 31 POCs, 10 were excluded because of MCC (around 30%) and one had major aneuploidy. CMA in POCs identified pathogenic copy number variations (CNVs) in 25% of cases (5/20) and variant of unknown significance (VUS) in 20% of cases (4/20). Autosomal trisomy was the most frequent chromosomal abnormality diagnosed. NGS was performed to establish single-gene causes of RPL. Couple exome sequencing was performed in 20 couples, and 14 were found to be carriers for autosomal recessive conditions. A total of 50 potential disease-causing variants in 40 genes were identified in 33 of 40 individuals (82.5%). Putative causative variants were identified in 37.5% of the TRIO cases (3/8). Mutations in few important genes (*SRP54, ERBB4, NEB, ALMS, ALAD, MTHFR, F5,* and *APOE*), which are involved in vital pathways, early embryonic development, and fetal demise, were identified in the POCs.

**Conclusion:** It enhances our understanding of prenatal phenotypes of many Mendelian disorders. These mutated genes may play an auxiliary role in the development of treatment strategies for RPL. There was no correlation of the number of abortions with etiological yield of any technique to detect the cause of RPL. This study shows the utilization of combination of techniques in improving our understanding of the cause of early embryonic lethality in humans.

## 1 Introduction

Recurrent pregnancy loss (RPL) is defined as two or more clinical pregnancy losses before 20 weeks of gestation (Practice committee, 2020). It affects 2–5% of couples trying to conceive (Ford and Schust, 2009). RPL can culminate into bad obstetric history (BOH), which implies previous unfavorable fetal outcome in terms of two or more consecutive spontaneous abortions, early neonatal deaths, still births, intrauterine fetal deaths, intrauterine growth retardation, and congenital anomalies (Singh and Sidhu, 2010). It is a major health concern and sometimes misconsidered as infertility. RPL is an emotionally challenging and a taxing condition for the couples, especially for maternal health issues. If a pregnancy is delayed or not successful because of any reason, the available reproductive years are shortened, resulting in a sense of urgency to conceive. Advanced maternal age is one of the highly associated factors with miscarriage (Gardner and Sutherland, 2004). It is very important to find out the reason behind miscarriage so that subsequent measures could to be taken to avoid the recurrence of such situation. Genetic factors such as chromosomal anomalies, particularly balanced translocations, are the major cause (approximately 5% prevalence in couples) for RPL (Ford and Schust, 2009). This prevalence is significantly higher than the normal population (∼1 in 500) (Jacobs et al., 1974).

A major number of couples with RPL (41.4%) have chromosomal abnormality, which can be picked up by karyotyping (Sugiura-Ogasawara et al., 2012). Chromosomal imbalances in gametes, resulting from balanced translocation in one of the couples, give rise to systemic errors in mitotic process during the early developmental stage of the conceptus. In about 50% of the couples with RPL, the underlying cause remains unsolved (Christiansen et al., 1990), although there might be involvement of genetic factors. The challenging key question in investigating the inherited predisposition to RPL is to decide the study subject in RPL (Rull et al., 2012). People have used conventional karyotyping and array-based comparative genomic hybridization in couples with RPL. Little focus has been given on the use of whole-exome/-genome sequencing in lethal *in utero* fetal disorders. Recent studies showed there are few gene sequence variations in couples with the history of RPL with or without their abortus that might contribute to RPL or related lethal birth defects (Quintero-Ronderos et al., 2017; Najafi et al., 2021; Xiang et al., 2021).

The genetic studies designed to explore the genes and mechanism behind pathogenesis of RPL have several challenges and there have been no consistent results due to variations in the selection of study subjects (couples with a history of RPL or female patients, or product of conception (POC) and controls. Due to complexities in collecting and processing the clinical samples from couples with a history of RPL and pregnancy loss events, there are limited genetic studies on the RPL families. In this study, both the genome of the parents and POC were examined. POCs were analyzed using CMA, and the cases without abnormal CNVs were subjected to TRIO exome analysis. India being a densely populated and diverse country with varied ethnicity and marriages in blood relations, we assumed that we would get certain novel autosomal recessive conditions. In this study, we have targeted a less explored area of genetics, especially in the Indian scenario to find out the genetic etiology of RPL; some of them undescribed previously. Apart from this, we have found the contribution of chromosomal and single gene disorders give rise to pregnancy loss, many a times *de novo* and at times inherited. This approach can help identify more genes core to human development and causative of RPL. The information from RPL testing can be helpful for patients and physicians to understand the cause of miscarriage and subsequently to develop a plan to support a successful future pregnancy.

## 2 Methodology

### 2.1 Study design

A multicenter prospective study was carried out over a period of 2 years (2020–2022). Medical genetic centers from two different tertiary care hospitals in North India participated in the study. Ethical clearance was taken from the ethics committees of both Institutes. All experimental protocols were approved by the Institutional Ethical Committees.

### 2.2 Subjects and sample size

It was a descriptive pilot study, so the sample size was not calculated. This study included 61 consecutive couples (122 individuals) with the history of RPL who visited the OPD/IPD of any of the two medical genetics centers during the miscarriage (POC samples were obtained) or after the miscarriage (genetic counselling for future pregnancy). An informed consent for testing under research and publication of data was taken as per the protocol.

### 2.3 Clinical details

A detailed clinical history was obtained from each patient, which included their obstetric history at present gestation, gestational duration in previous miscarriages, and family history of RPL (if any). All couples were evaluated for systemic illnesses, and their previous medical history was taken.

### 2.4 Samples

#### 2.4.1 Products of conception

POC samples sent from other centers and in-house in two centers were included. In-house, POCs were collected freshly in normal saline with aseptic precautions. All POC samples were checked for quality. Highly putrefied samples and samples collected in formalin were discarded. The remaining samples were checked under a microscope for chorionic villi or fetal tissue. Of the 61 samples, eight samples were putrefied, 14 came in formalin, and in eight samples, no chronic villi or fetal tissue could be obtained. Only 31 were processed further. In apparently 12 freshly obtained POC samples with good chorionic villi, culture was initiated for fetal karyotyping and obtaining fetal cells. For all 31 POC samples, which were selected for further processing, DNA was extracted from part of the sample using the QIAamp DNA Tissue extraction kit (QIAGEN, Hilden, Germany). Extracted DNA was quantified spectrophotometrically at 260 nm using Nanodrop (Thermo Nanodrop 2000), and the quality of DNA was assessed at 260/280 nm ≤ 1.8.

#### 2.4.2 Peripheral blood samples

Blood samples of couples were collected into the heparin tube and EDTA tube (2 mL each) from the couples for diagnostic chromosome and molecular analysis, testing for maternal cell contamination (MCC) in POC and further testing. DNA from peripheral blood samples was extracted using QIAGEN kits as per the manufacturer’s guidelines. Extracted DNA quality and quantity were checked using the Nanodrop instrument for further downstream processing.

### 2.5 QF-PCR in POC samples

QF-PCR was performed using the Devyser Compact v3 kit, and the markers specific to the chromosomes 13, 18, 21, X, and Y were amplified (Torres et al., 2021). The amplified markers were then subjected to capillary electrophoresis. The data were analyzed using GeneMapperTM (Thermofisher) and interpreted to determine the MCC and aneuploidy of fetus for specific chromosomes. QF-PCR in POC samples with markers corroborated with that of the mothers was carried out to check for MCC. Before proceeding with cytogenetic microarray and/or exome sequencing in POC, MCC and major chromosomal aneuploidies in 13, 18, 21, and sex chromosomes were ruled out.

### 2.6 Karyotyping in couples

From blood collected in heparin, karyotyping of couples has been carried out. G-banded karyotyping was carried out using trypsin–Giemsa banding preparations.

### 2.7 Other tests for RPL

All female partners were examined for thrombophilia (factor V Leiden, prothrombin G20210A gene mutation), antiphospholipid antibodies (APLA), lupus anticoagulant, anticardiolipin antibodies, anti-β_2_-glycoprotein I antibodies, hormonal status (thyroid stimulating hormone (TSH), T3, T4, and prolactin), and ultrasonography for uterine anomalies.

### 2.8 Multiplex ligation-dependent probe amplification (MLPA) in POC samples

MLPA for subtelomeric region was performed using SALSA MLPA Probemix P036 Subtelomeres Mix 1(MRC Holland) as per the manufacturer’s protocol.

### 2.9 Chromosomal microarray (CMA) in POC samples

CMA was performed using the Affymetrix 750K array Cytoscan kit in DNA samples extracted from POC samples as per the manufacturer’s recommendations. The Affymetrix 750K array Cytoscan kit has more than 750,000 markers (including 200,000 SNPs and 550,000 non-polymorphic probes).

Data were analyzed using ChAS software (Thermofisher Scientific, MA, United States) using a UCSC genome browser based on the GRCh38 genome assembly. Analysis of copy number variations (CNVs) was carried out using various tools and software like DECIPHER (https://decipher.sanger.ac.uk), DGV (http://dgv.tcag.ca/dgv), OMIM (www.omim.org), and ClinVar (https://www.ncbi.nlm.nih.gov/clinvar/). The interpretation of CNVs was based on recommended ACMG guidelines (South et al., 2013).

### 2.10 Next-generation sequencing

NGS was carried out using the Illumina HiSeq X10 platform (Illumina, Inc., San Diego, CA, United States). Briefly, DNA was sheared to produce 150–250 bp fragments for library preparation. Hybridization was carried out using the whole-exome ∼67 Mb Agilent Sure Select Clinical Research Exome V2 capture kit, following size selection, end-repair, phosphorylation, and adapter ligation to the DNA fragments according to the manufacturer’s protocol. Exome Library QC was checked on a Bioanalyzer (Agilent, United States) and quantified using Qubit (Invitrogen, United States). The libraries were sequenced as paired-end reads (2 × 150) for ∼80–100× coverage on HiSeq X (Illumina, CA). Couple exome or TRIO exome was carried out as per the availability of POC tissue and DNA quality.

#### 2.10.1 Data analysis

Sequence reads were aligned to human genome assembly GRCH37 using the Burrows–Wheeler Aligner (BWA) with the MEM algorithm. Variant calling and data processing were carried out using the Genome Analysis Toolkit (GATK, version 4.0,4.0, Broad Institute, https://software.broadinstitute.org/gatk/). Variant annotation was performed using ANNOVAR software (http://annovar.openbioinformatics.org/en/latest/). Filtering criteria used to narrow down the search of the causative variant: Synonymous, non-frameshift, or unknown variants, and low-quality reads were removed; frequency cut-off for population databases was set as <1% in dbSNP (http://www.ncbi.nlm.nih.gov/snp/), 1000 Genomes (http://browser.1000genomes. org/index.html), ESP6500 (http://evs.gs.washington.edu/EVS/), Exome Aggregation Consortium (ExAC; http://exac.broadinstitute.org/), and the Genome Aggregation Database (gnomAD; http://gnomad.broadinstitute.org/). Insilico analysis tools: PolyPhen2 (http://genetics.bwh. harvard.edu/pph2/), SIFT (http://sift.jcvi.org/), MutationTaster (http://mutationtaster.org/), and FATHMM-MKL (http://fathmm.biocompute.org.uk/fathmmMKL.htm) were used for variant effect prediction. ClinVar (ncbi.nlm.nih.gov/clinvar/), Human Genome Mutation Database: HGMD (https://www.hgmd.cf.ac.uk/ac/all.php), and DECIPHER database (https://decipher.sanger.ac.uk/) were used for identifying the reported or known SNVs and CNVs and the associated phenotypes.

#### 2.10.2 Validation of variants

Few of the putative variants identified by exome sequencing were confirmed by targeted sequencing and mutation analysis by polymerase chain reaction (PCR), followed by Sanger sequencing of the amplicon using BIG Dye Terminator (Applied Biosystems 3130 Genetic Analyzer; Applied Biosystems, Foster City, CA, United States). The raw data obtained were subsequently analyzed for the nucleotide variants (Supplementary Figure S2B).

### 2.11 Statistical analysis

Pearson correlation coefficient was applied in couples with different positive results and their experience of number of miscarriages in an attempt to find out if there is any effect of certain causes on number of abortions, and significance of the result was tested using online tool Statistics Kingdom (https://www.statskingdom.com/).

## 3 Results

### 3.1 Demography

In this study, the mean age of women was 29.9 years (25–40) and the mean age of men was 33.5 years (27–44). In two out of 61 (3%) couples, systemic illnesses, i.e., myotonic dystrophy (RPL52) and systemic lupus erythematosus (RPL34), were the probable causes of RPL (Supplementary Table S1). However, their POC samples were collected but could not be processed further due to putrefied tissue and MCC, respectively. No other obvious genetic or non-genetic causes were identified as the etiology of RPL in the remaining couples during the initial evaluation for endocrine causes and conditions leading to thrombophilia. While evaluating the past history, in two women, where previous POCs were subjected to genetic analysis, some abnormalities were found. They were, however, unrelated to the causes of RPL, i.e., one had monosomy X (POC9) and the other had trisomy 13 (POC8) (both detected by subtelomeric MLPA) (Supplementary Table S1). In our cohort, the number of women with previous two miscarriages was 12, three miscarriages in 27 women, four miscarriages in 10 women, five miscarriages in seven women, and ≥ 6 miscarriages in five women. We have tried to find if there was any correlation with the number of abortions and positive yield of chromosomal aberrations in POC (detected either by karyotype/QFPCR/MLPA/CMA), karyotype of couple, variations in the same gene for autosomal recessive conditions detectable by couple exome sequencing, or a positive result in trio exome. There was no significant correlation found with chromosomal aberrations in the POC and also with abnormalities detected by couple karyotype (Table 1). Women did not have any uterine malformations.

**TABLE 1:** Correlation of the number of abortions with a positive yield of different techniques.

Parameter	Value	Value	Value	Value
	X: Number of abortions Y: Positive results in POC QF-PCR or CMA	X: Number of abortions Y: Positive results in the couple karyotype	X: Number of abortions Y: Positive results of sequence changes in the same gene for AR conditions by the couple exome	X: Number of abortions Y: Positive results in the trio exome
Pearson correlation coefficient (r)	0.2851	0.1123	−0.137	−0.207
*p*-value	0.2103	0.3888	0.5659	0.6233
Covariance	0.1524	0.0459	−0.105	−0.089
Sample size (n)	21	61	20	8
Statistic	1.2967	0.8682	−0.585	−0.518
Interpretation	Results of the Pearson correlation indicated that there is a non-significant small positive relationship between X and Y (*r*(19) = .285, *p* = .210).	Results of the Pearson correlation indicated that there is a non-significant small positive relationship between X and Y (*r*(59) = .112, *p* = .389).	Results of the Pearson correlation indicated that there is a non-significant very small negative relationship between X and Y (*r*(18) = .137, *p* = .566).	Results of the Pearson correlation indicated that there is a non-significant very small negative relationship between X and Y (*r*(6) = .207, *p* = .623).

### 3.2 QF PCR in POC samples

Among 61 POCs, 50% (31/61) POCs were suitable for further processing. However, for the downstream processing (CMA and NGS), only 20 POCs were processed. In one sample (POC6), chromosomal aneuploidy trisomy 21 was detected by QF-PCR. The finding was corroborated by MLPA (subtelomeres) and by karyotyping. Ten samples were positive for MCC (Supplementary Table S1).

### 3.3 Multiplex ligation-dependent probe amplification (MLPA) in POC samples

MLPA was performed in nine samples where QF PCR ruled out maternal contamination. It confirmed trisomy 21 in one sample (POC6), which corroborated with QFPCR results and later on confirmed by karyotyping (Supplementary Table S1). This sample was not processed for cytogenetic microarray (CMA). In another sample, there was doubtful trisomy 11 (probe ratio 1.36 and 1.38 for p and q arms, respectively). This sample (POC39) was further processed for CMA, which confirmed trisomy 11 (Table 2).

**TABLE 2:** Details of CNVs detected in POCs.

ID	Type	Location	Size	No. of OMIM genes	Genomic co-ordinates	Interpretation	Comments
POC3	Loss	Chr17p11.2	775 Kb	4	arr[GRCh38]17p11.2(21,660,410_2,243,5981)x1	VUS	
POC4	Loss	Chr14q13.2	246 Kb	3	arr[GRCh38]14q13.2(34,823,799_35,070,625)x1	VUS	
POC5	Loss	Chr2q34	113 Kb	1	arr[GRCh38] 2q34(212,138,454_212,252,393)x1	VUS	
POC57	Gain	Chr7q36.3	1.7 Mb	5	arr[GRCh38] 7q36.3(157,546,042–159,327,017)x3	VUS	
POC18	Mosaic Loss	Chr19p13.3	47.9 Mb		arr[GRCh38] 19p13.3q13.33(260,912_ 8,250,320)x1 [0.26]	Likely pathogenic	Undetectable in the karyotype of the couple and likely to be *de novo*
	Loss	Chr17q24.2	1 Mb	9	arr[GRCh38] 17q24.2(67,202,603–68,276,036)x1		
POC23	Gain	Chr9p24.3	90.3 Mb	455	arr[GRCh38] 9p24.3q22.2(208,455_90,568,721)x3	Pathogenic	9:15 translocation detected in the male partner
	Loss	Chr15q11.2	21.3 Mb	339	arr[GRCh38] 15q11.2q15.3(22,582,283_43,964,261)X1	Pathogenic	
POC29	Loss	Chr11q24.3	6.8 Mb	50	arr[GRCh38] 11q24.3q25(128,253,364–135,067,522)x1	Pathogenic	
	Gain	Chr14	34.1 Mb	435	arr[GRCh38] 14q24.2q32.33(71,951,262–106,071,000)x3	Pathogenic	
POC33	Mosaic Gain	ChrX	155 Mb	-	arr[GRCh38] (X)x2 [0.30], (Y)x1	Pathogenic	
POC39	Gain	Trisomy Chr11	134 Mb	1671	arr[GRCh38] 11p15.5q25(230,615_134,938,470)x3	Pathogenic	

*VUS, variant of unknown significance.

### 3.4 Karyotyping of couples

**FIGURE 1:**
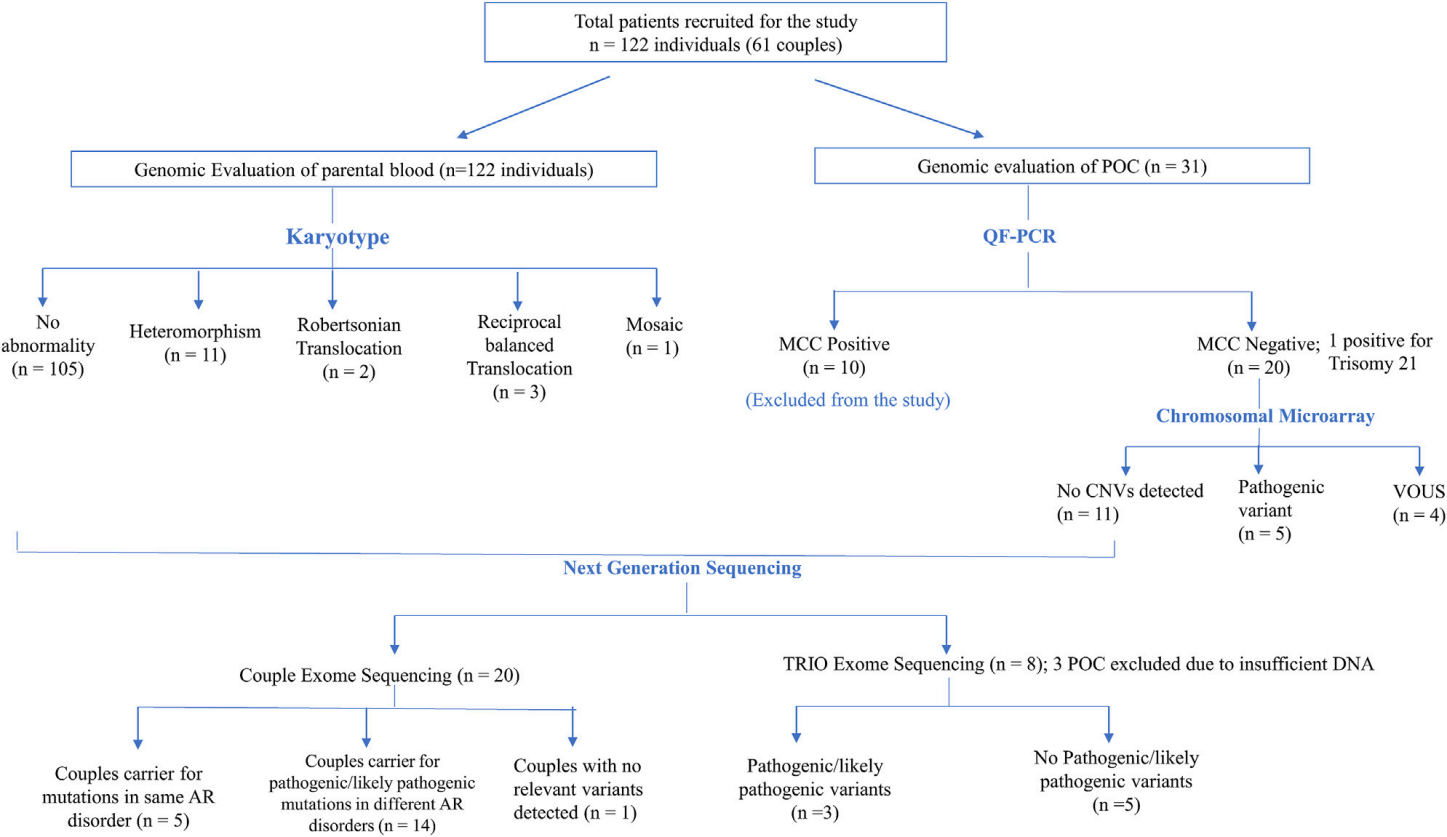
Flowchart depicting the details of samples analyzed in this study and summary of the CNVs and SNVs identified.

In six (5%) out of 122 individuals (61 couples), abnormality in karyotyping was detected (Table 3). Robertsonian translocation was present in two individuals and three were found to be balanced reciprocal translocation carriers. One individual was found to be mosaic for 46, XX and 46, XXX cell lines. The remaining 95% couples had normal karyotypes. Among 116 normal karyotypes, there were 11 heteromorphisms (9.5%), for which the couples were counselled and reassured (Figure 1). Out of the total, one rare type of heteromorphism pericentric inversion of chromosome Y was identified in an individual, which is reported to have no clinical significance (Verma et al., 1982).

**TABLE 3:** Karyotype data on couples with the history of recurrent pregnancy loss.

Chromosomal anomaly	Karyotype	Total no. of cases
Normal polymorphism		
Heteromorphism	46, XY, 9qh+	1
	46,XY, 9qh+,14ps+ & 21ps+	1
	46,XX,15ps, 21ps++	1
	46,XX,21ps+	1
	46,XY,21ps+	1
	46, XY,22ps+	1
	46,XY,13ps+,21ps+,22ps+	1
	46,XX,9qh+ & 22ps+	3
	46, X inv (Y) (p11.2q11.23)	1
Abnormal karyotype		
Balanced Robertsonian translocation	45, XX; rob (13,14) (q10:q10)	1
	45, XX, t (22:22) (q10; q10)	1
Balanced reciprocal translocation	46, XX, t (9; 15) (q22; q15)	1
	46, XX, t (5:15) (q23; q24)	1
	46, XY t (11:14) (q23; q31)	1
Mosaic	46, XX- 94% of cells and three cells showed karyotype of 47, XXX—6%	1

### 3.5 Karyotype from POC

Karyotype from POC was carried out for 12 samples, where good chorionic villi were obtained. These 12 samples were subjected to culture where eight showed good growth and in four, there was culture failure. Trisomy 21 was detected in one POC sample (POC6) (12%). In this pregnancy, cystic hygroma was detected in ultrasonography early in pregnancy (13 weeks).

### 3.6 Cytogenetic microarray

CMA in POCs identified pathogenic CNVs in 25% cases (5/20) and variant of unknown significance (VUS) CNVs in 20% cases (4/20) (Figure 1). In the remaining 55% cases, no significant CNVs were identified.

#### 3.6.1 Pathogenic CNVs

1. POC18 showed a low-level mosaic loss (26%) involving chromosome 19 (47.9 Mb) within region p13.3q13.33, indicating monosomy for this region (Supplementary Figure S1A). Monosomy of chromosome 19 is associated with pre- and postnatal growth retardation, psychomotor and language delay, hyperactivity, brachydactyly, hearing loss, anteverted nares, synophrys, hypodontia, and short neck. These were not detected in karyotype in either of the couple, suggesting it to be *de novo*. In addition, 1 Mb loss was detected on chromosome 17q24.2, indicating monosomy of this region, which included nine OMIM genes. Heterozygous loss of function variations of the *BPTF* gene is associated with delayed psychomotor development and intellectual disability, poor growth, small head size, dysmorphic facial features, and mild abnormalities of hands and feet.
2. POC23 showed a 90.3 Mb duplication of chromosome 9 and a deletion of 21.3 Mb on 15q11.2 (Supplementary Figure S1B). The observed duplication is consistent with partial trisomy 9. Partial trisomy of the short arm including the long arm of chromosome 9 is among the most common autosomal structural chromosomal anomalies.

CMA analysis showed another deletion spanning ∼21.3 Mb on chromosome 15q11.2-q15.3. This deletion overlaps with 15q deletion syndrome or distal 15q monosomy. Couple karyotyping showed 46,XX,t(9; 15) (q22; q15) in the female partner. CMA findings of duplication on chromosome 9 and deletion on chromosome 15 in POC corroborate with chromosomal translocation in couples at these loci.

3. In POC 29, CMA analysis showed a deletion spanning about ∼6.8 Mb on chromosome 11q24.3q25, including 50 genes. Deletion on 11q24.3q25 overlaps with 11q deletion syndrome, also known as Jacobsen syndrome (JBS: OMIM 147791) critical region. *ETS1* and *FLN1* are critical genes that have been proposed for causing Jacobsen syndrome phenotypes. Furthermore, the *FLN1* gene was present in the deleted region of the current case.

CMA analysis also showed a duplication spanning about 34.1 Mb on chromosome 14q24.2q32.33, including 435 genes. The observed duplication overlaps with a partial trisomy of 14q. In the submitted sample, the deletion on chromosome 11q and duplication on chromosome 14q suggest chromosomal rearrangement at these loci, such as translocation. The male partner was found to be a carrier of balanced translocation 46, XY,t(11:14) (q23; q31) (Supplementary Figure S1C).

4. POC33, CMA analysis showed a mosaic gain (∼30%) involving all SNPs on chrX (155 Mb) from Xpter to qter along with a single copy of Y-chromosome, indicating a rare form of Klinefelter syndrome. This condition is known as mosaic Klinefelter syndrome and individuals with this condition can have milder signs and symptoms than others with the XXY condition. Karyotypes of couples were normal (Supplementary Figure S1D).
5. POC39, the CMA analysis showed complete duplication on chromosome 11 spanning the entire chromosome (Supplementary Figure S1E). This duplication is consistent with complete trisomy 11. Complete trisomy 11 is an extremely rare phenomenon, and very little is known regarding the possible phenotypic features, as most cases of complete trisomy 11 are usually lethal and spontaneously aborted in the first trimester. The couple had a previous baby with developmental delay, whose karyotype, subtelomeric MLPA, and CMA were normal. Karyotypes of couples were also normal. Trisomy 11 in this instance is likely to be a *de novo* event.

#### 3.6.2 VUS CNVs

1. One couple (RPL3) was presented with a history of previous three abortions in the first trimester. Karyotypes of the couple were normal. POC3 CMA testing showed a loss at the cytoband region 17p11.2 spanning about 775 kbp (∼0.8 MB) encompassing four genes. There were two OMIM genes, *KCNJ18* and *PRG4*, present on the deleted segment. Variants in the *KCNJ18* gene have been found to be associated with thyrotoxic periodic paralysis-2 (TTPP2), but its haploinsufficiency association with the clinical phenotypes is yet to be identified. p53-responsive gene 4 (*PRG4*) overexpression (upregulated) in the apoptotic cells is mainly in association with *p53.* Since this deleted region has not been well established with syndromes or recurrent abortions and there is no established evidence in the literature, the observed finding has been labeled as VUS.
2. CMA of POC4 revealed a 247 Kb deletion on the chromosome 14q13.2 region, which includes three OMIM genes; *BAZ1A* (605680), *SRP54* (604857), and *FAM177A1* (619181). Among them, *SRP54* is associated with the autosomal dominant form of neutropenia (MIM# 618752).
3. CMA of POC5 showed heterozygous deletion of 113 Kb on chromosome 2q34, which includes only a single OMIM gene *ERBB4.*
4. CMA of POC57 identified a gain of 1.7 Mb on chromosome 7q36.3, which includes five OMIM genes. Clinical significance of the genes in the context of this patient’s phenotype is unknown.

### 3.7 Next-generation sequencing-based approaches

#### 3.7.1 Couple exomes

In the absence/inadequate POC DNA or in the case of MCC positive POC samples, couple exome sequencing was adopted in 20 couple samples. Out of the 20 couples, five couples (25%) were found to be a carrier of the mutation in the same genes (*PCNT, MTHFR, ALMS1, PKD1L1, NEB*, and *INPPL1*) causing autosomal recessive (AR) syndromes (Tables 4, 5). Fourteen couples (70%) were found to be carriers for pathogenic or likely pathogenic variants in other AR conditions (Table 6). No pathogenic or likely pathogenic variant was identified in one couple. According to the American College of Medical Genetics and Genomics guidelines (ACMG) (Richards et al., 2015), a total of 50 potential disease-causing variants in 40 genes were identified in 33 out of 40 individuals (82.5%), making them carriers of various AR disorders (Tables 4, 6).

**TABLE 4:** Couple exome (carrier status of genes which might explain the bad obstetric history: both partner carriers of mutations in the same gene for autosomal recessive disorders).

Couple ID	Gene	Wife	Husband	Disease
RPL2		W2	H2	
	*PCNT*	Status: CARRIER	Status: CARRIER	Microcephalicosteodysplastic primordial dwarfism type II (OMIM#210720)
		Variant: c.3710A>G (p.His1237Arg)	Variant: c.1334_1335del(p.Lys445ThrfsTer12)	Mode of inheritance: AR
		Zygosity: Heterozygous	Zygosity: Heterozygous	
		Classification: VUS (PM2 and BP4)	Classification: Likely pathogenic (PVS1 and PM2)	
		Accession: VCV000211856.7	Accession: SCV003804961	
RPL5		W5	H5	
	*MTHFR*	Status: CARRIER Variant: c.1286A>C(p.Glu429Ala)	Status: CARRIER	Homocystinuria due to methylene tetrahydrofolate reductase deficiency (OMIM#236250) Mode of inheritance: AR
		Zygosity: Heterozygous	Variant: c.1286A>C(p.Glu429Ala)	
		Classification: VUS (PP4 and BP6)	Zygosity: Heterozygous	
		Accession: VCV000003521.78	Classification: VUS (PP4 and BP6)	
			Accession: VCV000003521.78	
RPL16		W16	H16	
	*ALMS1*	Status: CARRIER	Status: CARRIER	Alstrom syndrome (OMIM#203800) Mode of inheritance: AR
		Variant: c.11734A>C(p.Ser3912Arg) Zygosity: Heterozygous	Variant: c.1420C>A(p.His474Asn) Zygosity: Heterozygous	
		Classification: VUS (PM2)	Classification: VUS (PM2 and BP4)	
		Accession: VCV000403949.6	Accession: VCV000459855.13	
RPL21		W21	H21	
	*PKD1L1*	Status: CARRIER	Status: CARRIER	Visceral heterotaxy-8 (OMIM#617205)
		Variant: c.8005C>T, (p.Arg2669Ter)	Variant: c.310G>A, (p.Ala104Thr)	Mode of inheritance: AR
		Zygosity: Heterozygous	Zygosity: Heterozygous	
		Classification: Pathogenic (PVS1, PM2, and PP5)	Classification: VUS (PM2) dbSNP: rs544795414	
		Accession: VCV001686969.1	Accession: SCV003806426	
RPL33		W33	H33	
	*NEB*	Status: CARRIER	Status: CARRIER	Arthrogryposis multiplex congenita 6 (OMIM#619334) Mode of inheritance: AR
		Variant: c.11706C>A (p.Asp3902Glu)	Variant: c.22454C>T (p.Thr7485Ile)	
		Zygosity: Heterozygous	Zygosity: Heterozygous	
		Classification: VUS (PM1+PM2+BP1)	Classification:	
			VUS (PM1+PM2+BP1)	
		Accession: SCV003804962	Accession: VCV000968876.7	
	*INPPL1*	Status: CARRIER	Status: CARRIER	Opsismodysplasia (258480)
		Variant: c.2839C>T(p.Pro947Ser) Zygosity: Heterozygous Classification: VUS (PM1+PM2+PP3)	Variant: c.3394G>A (p.Glu1132Lys)	Mode of inheritance: AR
		Accession: SCV003804963	Zygosity: Heterozygous	
			Classification: VUS (PM1+PM2+PP3)	
			Accession: VCV001391913.2	

*VUS, variant of unknown significance; AR, autosomal recessive.

**TABLE 5:** OMIM phenotypes related to mutated genes.

Gene	Associated disease/OMIM	Phenotype
*PCNT*	Microcephalic osteodysplastic primordial dwarfism type II (OMIM#210720)	MOPD II is characterized by intrauterine growth retardation, severe proportionate short stature, and microcephaly. It is distinct from Seckel syndrome (see 210,600) by more severe growth retardation, radiologic abnormalities, and absent or mild mental retardation
	Mode of inheritance: AR	
*MTHFR*	Homocystinuria due to methylene tetrahydrofolate reductase	Homocystinuria due to methylenetetrahydrofolate reductase deficiency is caused by homozygous or compound heterozygous mutation in the *MTHFR* gene on chromosome 1p36. Methylenetetrahydrofolate reductase deficiency is a common inborn error of folate metabolism. The phenotypic spectrum ranges from severe neurologic deterioration and early death to asymptomatic adults. In the classic form, both thermostable and thermolabile enzyme variants have been identified
	Deficiency (OMIM#236250)	
	Mode of inheritance: AR	
*ALMS1*	Alstrom syndrome (OMIM#203800)	Alstrom syndrome (ALMS) is caused by homozygous or compound heterozygous mutation in the *ALMS1* gene on chromosome 2p13. This disorder is characterized by progressive cone–rod dystrophy leading to blindness, sensorineural hearing loss, childhood obesity associated with hyperinsulinemia, and type 2 diabetes mellitus. Dilated cardiomyopathy occurs in approximately 70% of patients during infancy or adolescence. Renal failure and pulmonary, hepatic, and urologic dysfunction are often observed, and systemic fibrosis develops with age
	Mode of inheritance: AR	
*PKD1L1*	Visceral heterotaxy-8 (OMIM#617205)	Autosomal visceral heterotaxy-8 (HTX8) is an autosomal recessive developmental disorder characterized by visceral situs inverses associated with complex congenital heart malformations caused by defects in the normal left–right asymmetric positioning of internal organs.
	Mode of inheritance: AR	
*NEB*	Arthrogryposis multiplex congenita-6 (OMIM#619334)	Arthrogryposis multiplex congenita-6 (AMC6) is characterized by polyhydramnios and reduced fetal movements. Affected individuals have congenital joint contractures, dysmorphic facial features, distal skeletal anomalies with clenched hands and clubfeet, and edema with fetal hydrops. Fetal demise or termination of pregnancy often occurs after the ultrasound detection of abnormalities. Those that survive birth have significant hypotonia with absent spontaneous movements, respiratory insufficiency, arthrogryposis, and multiple pterygia. Skeletal muscle is hypoplastic, immature, and underdeveloped with nemaline rods, poorly developed sarcomeres, and poor cross-striation. Death in infancy usually occurs
	Nemaline myopathy-2 (NEM2)	
	Mode of inheritance: AR	
*INPPL1*	Opsismodysplasia (OMIM#258480)	Opsismodysplasia (OPSMD) is caused by homozygous or compound heterozygous mutation in the *INPPL1* gene on chromosome 11q13. This disorder is characterized by short limbs, small hands and feet, relative macrocephaly with a large anterior fontanel, and characteristic craniofacial abnormalities including a prominent brow, depressed nasal bridge, a small anteverted nose, and a relatively long philtrum. Death *in utero* or secondary to respiratory failure during the first few years of life has been reported, but there can be long-term survival. Typical radiographic findings include shortened long bones with delayed epiphyseal ossification, severe platyspondyly, metaphyseal cupping, and characteristic abnormalities of the metacarpals and phalanges
	Mode of inheritance: AR	

**TABLE 6:** Couple exome sequencing (incidentally detected important variations).

Patient ID	Gene	Variant	Zygosity	OMIM genes	Inheritance	Classification
W2	*CHST3*	c.533dup, p.Ala179ArgfsTer141	Heterozygous	Spondyloepiphyseal dysplasia with congenital joint dislocations (OMIM#143095)	AR	Pathogenic
		Accession: VCV000432012.15				
	*G6PD*	c.1003G>A, p.Ala335Thr	Heterozygous	Nonspherocytichemolyticanemia due to G6PD deficiency (OMIM#300908)	XLD	Pathogenic
		Accession: VCV000010363.35				
H2	*KIAA0556*	c.274_275del, p.Phe92LeufsTer23	Heterozygous	Joubert syndrome 26 (OMIM#616784)	AR	Pathogenic
		Accession: SCV003804999 rs1254671898				
H11	*ACADM*	c.486 + 1G>A	Heterozygous	Medium chain acyl-CoA dehydrogenase deficiency (OMIM# 201450)	AR	Pathogenic
		Accession: VCV000371544.9				
W11	*GNRHR*	c.785G>A (p.Arg262Gln)	Heterozygous	Hypogonadotropic hypogonadism-7 with or without anosmia (HH7) (OMIM# 146110)	AR	Likely pathogenic
		Accession: VCV000016024.20				
H16	*AMN*	c.1006 + 34_1007- 31del	Heterozygous	Imerslund–Grasbeck syndrome (OMIM# 618882)	AR	Pathogenic
		Accession: VCV000056742.4				
H21	*CFTR*	c.3209G>A, p.Arg1070Gln	Heterozygous	Cystic fibrosis (OMIM#219700)	AR	Pathogenic
		Accession: VCV000634835.1				
	*SLC24A5*	c.494C>G, p.Ser165Ter	Heterozygous	Oculocutaneous albinism-6 (OMIM#113750)	AR	Pathogenic
		Accession: SCV003804979				
	*RARS2*	c.1026G>A, p.Met342Ile	Heterozygous	Pontocerebellar hypoplasia type 6 (OMIM#611523)	AR	Likely pathogenic
		Accession: VCV000215064.20				
W21	*DOCK7*	c.1511_1513del, p.Pro504_Ter505delinsArg	Heterozygous	Fetal akinesia deformation sequence −3 (OMIM#618389)/Congenital myasthenic syndrome-10 (OMIM#254300)	AR	Pathogenic
		Accession: SCV003804980				
W35	*CFTR*	c.1367T>C, (p.Val456Ala)	Heterozygous	Cystic fibrosis (OMIM#219700)	AR	Pathogenic
		Accession: VCV000035821.37				
H35	*HBB*	c.27dupG (p.Ser10Valfs*14)	Heterozygous	Beta-thalassemia (OMIM# 613985)	AR	Pathogenic
		Accession: VCV000036308.80				
W37	*HBB*	c.27dupG (p.Ser10Valfs*14)	Heterozygous	Beta-thalassemia (OMIM# 613985)	AR	Pathogenic
		Accession: VCV000036308.80				
H37	*CFTR*	c.1367T>C (p.Val456Ala)	Heterozygous	Cystic fibrosis (OMIM#219700)	AR	Pathogenic
		Accession: VCV000035821.37				
	*ACADS*	c.136C>T (p.Arg46Trp) Accession: VCV000003825.12	Heterozygous	Acyl-CoA dehydrogenase, short chain, deficiency of (OMIM#201470)	AR	Pathogenic
	*ALOXE3*	c.1630C>T, (p.Gln544Ter) VCV000449286.4	Heterozygous	Congenital ichthyosis, 3 (OMIM# 606545)	AR	Pathogenic
	*VPS13A*	c.3740_3741insAGAG (p.Ser1249ArgfsTer11)	Heterozygous	Choreoacanthocytosis (OMIM#200150)	AR	Pathogenic
		Accession:				
		SCV003804981				
	*MKS1*	c.1450_1453dupGGCA (p.Thr485Argfs)	Heterozygous	Bardet–Biedl syndrome 13, Meckel Syndrome 1 (OMIM#615990)/Joubert syndrome 28 (OMIM# 617121)	AR	Pathogenic
		Accession:				
		SCV003804983				
W40	*IL11RA*	c.709C>T (p.Arg237Ter)	Heterozygous	Craniosynostosis and dental anomalies (OMIM#614188)	AR	Pathogenic
		Accession:				
		VCV001074686.3				
	*LMBRD1*	c.399delA (p.Lys133Asnfs*17)	Heterozygous	Methylmalonic aciduria and homocystinuria, cblF type (OMIM#277380)	AR	Likely pathogenic
		Accession:				
		SCV003804992				
	*PAH*	c.355C>T (p.Pro119Ser)	Heterozygous	Phenylketonuria (OMIM#261600)	AR	Likely pathogenic
		Accession:				
		VCV000092741.19				
W44	*CERKL*	c.316C>A, (p.Arg106Ser)	Heterozygous	Retinitis pigmentosa 26 (OMIM# 608380)	AR	Likely pathogenic
		Accession:				
		VCV000438054.10				
H44	*CEP63*	c.1833del, (p.Leu612Ter)	Heterozygous	Seckel syndrome 6 (OMIM# 614728)	AR	Pathogenic
		Accession:				
		SCV003804993				
W45	*ASPM*	c.2783del, (p.Ala928ValfsTer7)	Heterozygous	Primary microcephaly-5 (OMIM# 608716)	AR	Pathogenic
		Accession:				
		SCV003804994				
	*EPM2A*	c.179G>A, (p.Trp60Ter)	Heterozygous	Progressive myoclonic epilepsy-2 (OMIM#254780)	AR	Pathogenic
		Accession:				
		SCV003804995				
H45	*SH3TC2*	c.3511C>T, (p.Arg1171Cys)	Heterozygous	Charcot–Marie–Tooth disease type 4C (OMIM# 601596)	AR	Likely pathogenic
		Accession:				
		VCV000448370.30				
W48	*SLC6A19*	c.311G>A, (p.Trp104Ter)	Heterozygous	Hartnup disorder (OMIM#234500)	AR	Pathogenic
		Accession:				
		SCV003804998				
H48	*HADHA*	c.1195C>T, (p.Arg399Ter)	Heterozygous	Mitochondrial trifunctional protein deficiency (OMIM#609015)/LCHAD deficiency (OMIM#609016)	AR	Pathogenic
		Accession:				
		VCV000449455.12				
H53	*ABCA4*	c.5882G>A, p.Gly1961Glu	Heterozygous	Cone–rod dystrophy-3 (OMIM#604116)/Retinitis pigmentosa-19 (OMIM#601718)/Stargardt disease-1 (OMIM#248200)	AR	Pathogenic
		Accession:				
		VCV000007888.78				
W53	*PMPCB*	c.524G>A, p.Arg175His	Heterozygous	Multiple mitochondrial dysfunctions syndrome-6 (OMIM#617954)	AR	Likely pathogenic
		Accession:				
		VCV000523140.3				
	*MYO7A*	c.5345G>C, p.Gly1782Ala	Heterozygous	Deafness −2 (OMIM#600060)/Usher syndrome, type-1B (OMIM#276900)	AR	Likely pathogenic
		Accession:				
		VCV000560898.6				
H55	*CPLANE1*	c.3056_3059dupTGTG (p.Trp1020Cysfs*6) SUB12936984	Heterozygous	Joubert syndrome 17 (OMIM# 614615)/Orofaciodigital syndrome VI (OMIM#277170)	AR	Likely pathogenic
W55	*HYDIN*	c.12444-1G>A	Heterozygous	Ciliary dyskinesia, primary, 5 (OMIM# 608647)	AR	Pathogenic
		Accession:				
		SCV003836565				
H60	*G6PD*	c.563C>T (p.Ser188Phe)	Hemizygous	Glucose-6-phosphate dehydrogenase deficiency (OMIM#300908)	XLR	Likely pathogenic
		Accession:				
		VCV000100057.65				
H61	*GUCY2D*	c.849C>A, p.Tyr283Ter	Heterozygous	Choroidal dystrophy-1 (OMIM#215500)/Leber congenital amaurosis 1 (OMIM#204000)/Congenital stationary night blindness type 1I (OMIM#618555)	AR	Pathogenic
		Accession:				
		VCV000521653.4				
	*PTPRQ*	c.3308_3309del, p.Leu1103ArgfsTer4	Heterozygous	Deafness 84A (OMIM#613391)	AR	Pathogenic
		Accession:				
		SCV003804996				
W61	*F5*	c.1601G>A, p.Arg534Gln	Heterozygous	Factor V deficiency (OMIM#227400)	AR	Likely pathogenic
		Accession:				
		VCV000000642.85				
	*RTN4IP1*	c.59G>A, p.Trp20Ter	Heterozygous	Optic atrophy 10 with or without ataxia, mental retardation, and seizures (OMIM#616732)	AR	Pathogenic
		Accession:				
		VCV001405046.2				
	*GAS8*	c.495 + 1G>T	Heterozygous	Primary ciliary dyskinesia, 33 (OMIM#616726)	AR	Pathogenic
		Accession:				
		SCV003806425				
	*MARS1*	c.1793G>A, p.Arg598His	Heterozygous	Nonphotosensitive trichothiodystrophy-9 (OMIM#619692)/Interstitial lung and liver disease (OMIM#615486)	AR	Pathogenic
		Accession:				
		VCV001172763.3				

*AR, autosomal recessive; AD, autosomal dominant; XLD, X-linked dominant; XLR, X-linked recessive.

#### 3.7.2 Carrier for autosomal recessive Mendelian disorders

Five couples were identified with putative candidate gene variants in same gene qualifying them as carriers for autosomal recessive conditions (Table 5) which can be the putative causative variant in fetus (Table 4; Figure 1).

1. RPL2 couple: This RPL2 couple was found to be carriers of a likely pathogenic variant and a VUS in the *PCNT* gene (p.Lys445ThrfsTer12 and p.His1237Arg). The *PCNT* gene is causative of AR microcephalic osteodysplastic primordial dwarfism type II (OMIM#210720).
2. RPL5 couple: This RPL5 couple presented with a history of RPLs and hydrops. Both of them were found to be carriers for the same variant for methylenetetrahydrofolate reductase (*MTHFR*) deficiency. Sanger validation has been performed for this variant (Supplementary Figure S2Bi). This missense variant NM_005957.5 (*MTHFR*):c.1286A>C (p.Glu429Ala) has been reported in ClinVar as likely pathogenic (Accession: VCV000003521.78). The observed variant is a well-known polymorphism and has been observed with a high allele frequency in both 1000 Genomes and gnomAD databases. The reference base is conserved across the species, and *in silico* predictions by PolyPhen and SIFT are damaging. This variant has been reported to cause an increased risk of fetal abnormalities by Pezzetti et al. (2004). For these reasons, this variant has been classified as VUS likely to be pathogenic.
3. RPL16 couple: This non-consanguineously married couple, presented with history of three miscarriages and 4th pregnancy showed omphalocele on ultrasound at 14 weeks 4 days gestation age. On doing the couple exome, both of them were incidentally found to be carriers of sequence variations in the *ALMS1* gene, which is causative of Alstrom syndrome (ALMS). ALMS is caused by homozygous or compound heterozygous mutation in the *ALMS1* gene on chromosome 2p13. The missense variant NM_015120.4 (*ALMS1*):c.11734A>C (p.Ser3912Arg) was identified in the wife, which is reported as VUS in ClinVar (Accession: VCV000403949.6). The p.Ser3912Arg variant is observed in 6/30,604 (0.0196%) alleles from individuals of gnomAD South Asian background in gnomAD. Another missense variant c.1420C>A (p.His474Asn) in the same gene in the heterozygous state was detected in the spouse sample. This variant was reported as VUS in ClinVar (Accession: VCV000459855.13). The p.His474Asn variant is observed in 12/30,596 (0.0392%) alleles from individuals of gnomAD South Asian background in gnomAD. Both the missense variants were predicted to be damaging by both SIFT and PolyPhen2. For these reasons, these variants have been classified as VUS. Sanger validation has been performed for both the variants (Supplementary Figure S2Bii).
4. RPL21 couple: This couple was found to be carriers of VUS in the *PKD1L1* gene (p.Ala104Thr) and a pathogenic variant in the *PKD1L1* gene (p.Arg2669Ter), respectively. The homozygous variant in the *PKD1L1* gene is responsible for visceral heterotaxy-8 (OMIM#617205).
5. RPL33 couple: This non-consanguineously married couple presented with a history of five recurrent miscarriages. During 6th gestation, CMA of POC was indicative of mosaic Klinefelter syndrome. Couple karyotype was reported normal. Couple exome sequencing revealed the carrier status of the couple for two AR disorders.

The first disorder was arthrogryposis multiplex congenita-6 (AMC6)/Nemaline myopathy-2 (NEM2), which is caused by homozygous or compound heterozygous mutation in the *NEB* gene on chromosome 2q23. The wife was found to be a carrier of missense variant NM_001271208.2:c.22454C>T (p.Thr7485Ile) in the *NEB* gene. Another missense variant c.22454C>T (p.Thr7485Ile) in the same gene in the heterozygous state was detected in the spouse sample (Table 4). The missense variant c.22454C>T (p.Thr7485Ile) was found in ClinVar (Accession: VCV000968876.7) with a classification of VUS. This variant is observed in 11/30,602 (0.0359%) alleles from individuals of gnomAD South Asian background in gnomAD. The p.Asp3902Glu variant is novel (not in any individuals) in gnomAD. Both the variants were predicted to be damaging by both SIFT and PolyPhen2, and the mutant residues were conserved in all mammalian species. For these reasons, these variants have been classified as VUS.

Both were found to be carriers for another AR disorder: opsismodysplasia (OPSMD), which is caused by homozygous or compound heterozygous mutation in the *INPPL1* gene on chromosome 11q13. The husband was found to be a carrier for missense variant NM_001567.4 (*INPPL1*):c.3394G>A (p.Glu1132Lys), which is reported as VUS in ClinVar (Accession: VCV001391913.2). The p.Glu1132Lys variant is observed in 4/30,238 (0.0132%) alleles from individuals of gnomAD South Asian background in gnomAD.

Another missense novel variant in the same gene in the heterozygous state c.2839C>T (p.Pro947Ser) was detected in the spouse sample, which is not reported in any database. Both the missense variants were predicted to be damaging by both SIFT and PolyPhen2. The mutant residues were conserved in all mammalian species. For these reasons, this variant has been classified as VUS. Sanger validation has been performed for these variants (Supplementary Figure S2Biii).

#### 3.7.3 Other findings (incidentally detected important variations)

Other pathogenic and likely pathogenic variants identified in autosomal recessive disorders are given in Table 6. These variants were grouped into a separate table as both of the couple were not found to be carriers for the same genes. These variants were found in either of the couple in genes *HBB, CFTR, ACADS, ALOXE, VPS13A, MKS1, MTHFR, F5, ACADM, GMPPB, GNRHR, AMN, CERKL, CEP63, ASPM, EPM2A, SH3TC2, SLC6A1, HADHA, CHST3, G6PD, KIAA0556, PKD1L1, SLC24A5, RARS2, DOCK7, PKD1L1, CPLANE1, HYDIN, GUCY2D, PTPRQ, SLX4, RTN4IP1, GAS8, MARS1, ABCA4, PMPCB,* and *MYO7A* (Table 6).

#### 3.7.4 Trio exomes

Trio exome analysis was performed in eight trios to identify the genomic reason of unexplained RPL. Putative causative variants were identified in three trios (Table 7; Figure 1).

1. **RPL28:** Trio exome analysis showed a frameshift *de novo* variant c.413dup (p.Met138fs) in the *F5* gene. Susceptibility to RPL-1 (RPRGL1) (MIM#614389) is conferred by variation in the coagulation factor V gene. This variant was not present in parents. As per ACMG guidelines, this variant is predicted to be likely pathogenic (PVS1 and PM2).
2. **RPL27:** NGS Trio revealed a homozygous missense variant c.388T>C (p.Cys130Arg) in the *APOE* gene in fetus and parents were found to be carriers for the same. This variant is reported as pathogenic in ClinVar (Accession: VCV000441268.4). *APOE* has been shown to be associated with an elevated risk of recurrent miscarriage.
3. **RPL50:** RPL50 showed a homozygous missense variant NM_000031.6(*ALAD*):c.446G>A (p.Arg149Gln) in fetus, which has not been reported previously in the database. The p.Arg149Gln missense variant is predicted to be damaging by both SIFT and PolyPhen2. The arginine residue at codon 149 of *ALAD* is conserved in all mammalian species. For these reasons, this variant has been classified as VUS. The same variant in the heterozygous state was detected in the father and the mother, and Sanger validation has been performed for the same (Supplementary Figure S2A). Porphyria, acute hepatic, is caused by mutation in the gene encoding delta-aminolevulinate dehydratase on chromosome 9q32. The disorder is characterized by the failure to thrive, respiratory analysis, vomiting, abdominal colic, hypotonia, muscle weakness, hemolytic anemia, and porphyria. A study has showed that porphyria disorder is closely associated with pregnancy risk (Tollånes et al., 2011).

**TABLE 7:** Trio exome results.

ID	Gene	Variant	Zygosity (in POC)	OMIM disorder	Inheritance	Origin	Classification as per ACMG guidelines
RPL27	*APOE*	c.388T>C	Homozygous	Recurrent pregnancy loss susceptible gene	AR	Inherited	Likely pathogenic (PM2 and PP5)
		p.Cys130Arg					
		Accession: VCV000441268.4 (rs429358)					
RPL28	*F5*	c.413dup	Heterozygous	Thrombophilia OMIM# (188055)	AD	*De novo*	Likely pathogenic (PVS1 and PM2)
		p.Met138fs					
		Accession: SCV003804964					
RPL50	*ALAD*	c.446G>A (p.Arg149Gln)	Homozygous	Porphyria, acute hepatic (OMIM#612740)	AR	Inherited	VUS (PM2, PP2, and PP3)
		Accession: SCV003804978					

*AR, autosomal recessive; AD, autosomal dominant; VUS, variant of unknown significance.

## 4 Discussion

This present study represents an Indian series of complete cytogenetic and molecular analyses of the first-trimester pregnancy loss abortus (31 POCs) and further evaluation in couples (61 couples). The various laboratory techniques used were karyotyping, CMA, and couple/trio exome sequencing. Most miscarriages were in the first trimester, and we had little information on fetal anomalies or phenotypes except embryonic lethality. This made the analysis and interpretation more complex. From each family, we could get only one POC and could not get the samples from previous pregnancies to verify the variant and do the segregation analysis. Because of the dense Indian population and marriages within blood relations, we hypothesized that there would be detection of some autosomal recessive Mendelian disorders in fetuses. There can be early embryonic lethality in such conditions, and in view of the absence of detailed prenatal phenotypes and associated laboratory test reports, some of the causes are often missed (Jelin and Vora, 2018).

Cytogenetic analysis of POCs is important to get a clue of the cause of fetal lethality/loss. This information can be used to estimate the risks of recurrence in future pregnancies. From India, a study by Dutta et al. (2011) showed majority of the cases of RPL was having balanced reciprocal translocations (Dutta et al., 2011). In our study, the most frequent chromosome abnormalities diagnosed in fetuses were autosomal trisomy and double segment imbalances (DSIs). Recently, Gajjar et al., showed causative CNVs in 38% of the POC samples (Gajjar et al., 2023) while pathogenic CNVs were identified in 25% of the POCs in our study.

Among chromosomal aberrations in fetuses, trisomy 9 was detected in a fetus (POC23) along with monosomy of 15q11.2. Trisomy 9 is a rare and often fatal chromosomal abnormality which occurs in approximately 2.4% of pregnancy losses (Miryounesi et al., 2016). Clinical phenotypes are craniofacial dysmorphism including hypertelorism, prominent nose, deep-set eyes, and down-slanting palpebral fissures (Temtamy et al., 2007). The specific symptoms of partial trisomy 9 are varied depending on the size of duplication on chromosome 9p and 9q. 15q11.2 microdeletion syndrome is a relatively rare chromosomal abnormality, which is characterized by pre- and postnatal growth restriction, developmental delay, variable degrees of intellectual disability, hand and foot anomalies, and mild craniofacial dysmorphism (Rudaks et al., 2011). The reports of 15q11.2 microdeletion on prenatal ultrasonographic abnormalities are rare. Other features are neonatal lymphedema, heart malformations, aplasia cutis congenita, aortic root dilatation, and autistic spectrum disorder have also been reported (Pinson et al., 2005). POC29 revealed double segment imbalance deletion at chromosome 11q24.3 and duplication of 14q24.2q32.33. Both are terminal, and the underlying cause was balanced translocation in any one partner of the couple. Deletion at chromosome 11q24.3 is linked with the Jacobsen syndrome critical region. Reported patients had pancytopenia of variable degrees (including thrombocytopenia) and neonatal bleeding (Serra et al., 2021). Fetal growth retardation, low birth weight, and perinatal asphyxia are risk factors which have contributed to variable clinical severity (Ichimiya et al., 2018). Trisomy 11 was detected in one fetus (POC39). Full trisomy 11 has not been reported in live births and presumably leads to early pregnancy loss (Balasubramanian et al., 2011). Few cases with partial trisomy 14q were reported with carrying pericentric inversion in one of the parents and proband with recombinant chromosome 14 (Kurtulgan et al., 2015).

Among VUS CNVs, few important genes were identified. In POC5, a small deletion in 2q34 was identified, which encompasses a gene *ERBB4* that is an important receptor in the control of fetal lung type II cell maturation (Zscheppang et al., 2007). Downregulation of the *ERBB4* gene is responsible for insufficient fetal surfactant production which further leads to respiratory distress syndrome in preterm infants (Zscheppang et al., 2007).

Couples with a karyotype in one of the partners showing heteromorphisms were counselled that they were normal variations; counselling could alleviate their anxiety and guilt, somewhat helping them to cope with the mishap. Similarly, POCs showing non-recurrent causes like trisomy 21 in POC6 and trisomy 11 in POC39 helped in finding a definitive cause at least for that loss and help in making them understand that chromosomal and genetic defects might be acquired *de novo* and there remains a fair chance of normal natural pregnancy outcome in future pregnancies. During follow-up in RPL6, we observed that there was a successful natural pregnancy and the baby is about 8 months now. They had undergone prenatal testing at 16 weeks for Down syndrome in view of the previously detected trisomy 21 in POC6.

Doing NGS on couples with the history of RPL and POCs is an efficient approach to identify the lethal genes and genes essential for embryonic development. Additionally, it gives the advantage of identifying certain prenatal phenotypes of many Mendelian conditions. In our study, 98% of variants were autosomal recessive. In the absence of the POC sample, couple exome sequencing was carried out. Carrier couples (RPL33) were identified with the variants in the *NEB* gene, which is responsible for AR Arthrogryposis multiplex congenita-6 (AMC6). Clinical features of AMC6 include congenital joint contractures, facial dysmorphism, skeletal anomalies, and edema with fetal hydrops. Death in infancy or fetal demise usually occurs (Ahmed et al., 2015; Rocha et al., 2021). This couple was also found to be carriers for mutation in the *INPPL1* gene, which is responsible for AR opsismodysplasia (OPSMD). No anomalies could be detected in our case because of early miscarriage.

Other AR gene *ALMS* variants were identified in couples with the history of RPL (RP16). Dilated cardiomyopathy occurs in approximately 70% of patients during infancy or adolescence (Marshall et al., 1997; Collin et al., 2002). Severe form of this disorder can be the cause of RPL in this couple.

RPL5 couple was found to be carriers for mutation in the *MTHFR* gene. It is already known that women with a *MTHFR* variant have a higher risk for pregnancy-related issues such as miscarriages, preeclampsia, or a baby born with birth defects, such as spina bifida. The theory behind the connection between the *MTHFR* mutation and pregnancy loss is that tiny blood clots are formed because of homocysteinemia, which blocks the flow of nutrition to the placenta, essentially starving the fetus and triggering a spontaneous abortion (Dell’dera et al., 2018). Another gene in the list was *PCNT* causing MOPD II (Couple RPL2), which is characterized by intrauterine growth retardation, severe proportionate short stature, and microcephaly. This can be the cause of fetal demise in this case.

Interestingly, in trio exome (RPL50), fetus was found to harbor homozygous variant in the *ALAD* gene which is responsible for porphyria, acute hepatic. Both parents were found to be carriers for the same. Fujita et al., 1987 reported a case with severe infantile-onset of acute hepatic porphyria (612,740), where the couple also had four successive pregnancy losses (Fujita et al., 1987). We have found *de novo* likely pathogenic variant the *F5* gene in fetus (RPL28). This gene encodes a protein called coagulation factor V, which plays a critical role in the coagulation system. In response to injury, it leads to a series of chemical reactions that forms blood clots. Pregnancy failure is often known to be associated with mutations that promote thrombophilia (Hansda and Roychowdhury, 2012). There are many supporting evidence that women with thrombophilia have higher risk of pregnancy-related venous thromboembolism and possibly other pregnancy-related issues such as pregnancy loss (Calderwood and Greer, 2005). We have found a homozygous variant in fetus, which was inherited from parents (RPL27). Research studies have showed a relationship between RPL and apolipoprotein E (Apo E) gene polymorphisms (Li et al., 2014).

By doing the carrier screening for various AR disorders, especially for pathogenic/likely pathogenic variants, we got additional information about the carrier status of various common (thalassemia, cystic fibrosis, and G6PD deficiency) and rare disorders (Table 6). Based on the carrier status of the couple and knowledge of the lethal genes involved in embryo development, it is important to counsel the families regarding risks of recurrence and future pregnancy options (Soden et al., 2014; Alamillo et al., 2015).

Early embryonic lethality leads to the termination of pregnancy. The causes being many ranging from maternal illness, *de novo* or inherited chromosomal anomalies in the embryo to various single gene disorders, sometimes inherited in autosomal recessive or X-linked manner from healthy parents, and sometimes as new autosomal dominant lethal mutations during gametogenesis. Our study aimed to find a systemic approach with a combination of techniques which can be carried out to understand the underlying cause. With a very high chance of insufficient/poor quality tissue of POC and maternal contamination, it is probably a good practice to obtain tissue antenatally after consent: as the couple RPL6 who opted for the collection of antenatal tissue before MTP lest good quality sample could not be obtained after termination. We could obtain the result by karyotyping and QF-PCR only. In the case of POC29, once the POC was found suitable, with negative MCC report, CMA was performed, which revealed double segment imbalance involving chromosomes 11 and 14 and male partner in the couple was found to be carrier of balanced translocation involving the aforementioned chromosomes. The cases where no chromosomal aberrations were found were subjected to trio exome or couple exome and causative variants were identified in some as in couple RPL 33. However, good POC samples could not be obtained in a huge number of couples and we directly went ahead with couple exome. In case of uncertainties, a combination of tests had been definitely useful.

With the advent of robust tools for sequencing the genome, various researchers have been trying to find novel genes using exome sequencing of the couple or trio to explain the underlying causes of RPLs (Quintero-Ronderos et al., 2017; Xiang et al., 2021). Although there had been some genes which had been recurrently implicated, the etiology remains unexplained in a very big proportion of couples. In our study, we used exome sequencing in a very small number of couples/trio and that remains the major caveat of our study. Nevertheless, we were able to highlight the importance of genetic testing in the POC in spite of difficulties in obtaining good samples and a very high failure rate of testing. A systematic approach and redefining testing types would be helpful in finding the cause in the increasing number of couples and thereby would help in counselling for future pregnancies.

## Data Availability

The datasets presented in this study are available in the ClinVar, corresponding accession numbers are given in the tables. Novel variants have been submitted and can be found here: https://www.ncbi.nlm.nih.gov/clinvar/submitters/507431/.

## References

[B1] Ahmed A. A. Skaria P. Safina N. P. Thiffault I. Kats A. Taboada E. (2015). Arthrogryposis and pterygia as lethal end manifestations of genetically defined congenital myopathies. Am. J. Med. Genet. 176A, 359–367. 10.1002/ajmg.a.38577 29274205

[B2] Alamillo C. L. Powis Z. Farwell K. Shahmirzadi L. Weltmer E. C. Turocy J. (2015). Exome sequencing positively identified relevant alterations in more than half of cases with an indication of prenatal ultrasound anomalies. Prenat. Diagn 35 (11), 1073–1078. 10.1002/pd.4648 26147564

[B38] Balasubramanian M. Peres L. C. Pelly D. (2011). Mosaic trisomy 11 in a fetus with bilateral renal agenesis: co-incidence or new association?. Clin. Dysmorphol. 20 (1), 47–49. 10.1097/MCD.0b013e32833ff2e9 20966746

[B3] Calderwood C. J. Greer I. A. (2005). The role of factor V Leiden in maternal health and the outcome of pregnancy. Curr. Drug Targets 6 (5), 567–576. 10.2174/1389450054546024 16026277

[B4] Christiansen O. B. Mathiesen O. Lauritsen J. G. Grunnet N. (1990). Idiopathic recurrent spontaneous abortion. Evidence of a familial predisposition. Acta Obstet. Gynecol. Scand. 69, 597–601. 10.3109/00016349009028702 2094140

[B5] Collin G. B. Marshall J. D. Ikeda A. So W. V. Russell-Eggitt I. Maffei P. (2002). Mutations in ALMS1 cause obesity, type 2 diabetes and neurosensory degeneration in Alström syndrome. Nat. Genet. 31, 74–78. 10.1038/ng867 11941369

[B6] Dell'’dera D. L'’piscopia A. Simone F. Lupo M. G. Epifania A. A. Allegretti A. (2018). Methylenetetrahydrofolate reductase gene C677T and A1298C polymorphisms and susceptibility to recurrent pregnancy loss. Biomed. Rep. 8 (2), 172–175. 10.3892/br.2018.1039 29435277 PMC5778916

[B7] Dutta U. R. Rajitha P. Pidugu V. K. Dalal A. B. (2011). Cytogenetic abnormalities in 1162 couples with recurrent miscarriages in southern region of India: Report and review. J. assisted reproduction Genet. 28 (2), 145–149. 10.1007/s10815-010-9492-6 PMC3059523 20931274

[B8] Ford H. B. Schust D. J. (2009). Recurrent pregnancy loss: Etiology, diagnosis, and therapy. Rev. Obstet. Gynecol. 2, 76–83. 19609401 PMC2709325

[B9] Fujita H. Sassa S. Lundgren J. Holmberg L. Thunell S. Kappas A. (1987). Enzymatic defect in a child with hereditary hepatic porphyria due to homozygous delta-amino levulinic acid dehydratase deficiency: Immunochemical studies. Pediatrics 80 (6), 880–885. 3684400

[B10] Gajjar K. Patel A. Patel B. Chettiar S. Jhala D. (2023). Array comparative genomic hybridization analysis of products of conception in recurrent pregnancy loss for specific anomalies detected by USG. Reprod. Fertil. 4, 22–0092. 10.1530/RAF-22-0092 PMC10160560 36961397

[B11] Gardner M. R. J. Sutherland G. R. (2004). Chromosome abnormalities and genetic counseling. Oxford University press, 368.

[B12] Hansda J. Roychowdhury J. (2012). Study of thrombophilia in recurrent pregnancy loss. J. Obstet. Gynaecol. India 62 (5), 536–540. 10.1007/s13224-012-0197-x 24082554 PMC3526698

[B13] Ichimiya Y. Wada Y. Kunishima S. Tsukamoto K. Kosaki R. Sago H. (2018). 11q23 deletion syndrome (jacobsen syndrome) with severe bleeding: A case report. J. Med. Case Rep. 12 (1), 3. 10.1186/s13256-017-1535-5 29307309 PMC5757304

[B14] Jacobs P. A. Melville M. Ratcliffe S. Keay A. J. Syme J. (1974). A cytogenetic survey of 11,680 newborn infants. Ann. Hum. Genet. 37 (4), 359–376. 10.1111/j.1469-1809.1974.tb01843.x 4277977

[B15] Jelin A. C. Vora N. (2018). Whole exome sequencing: Applications in prenatal genetics. Obstet. Gynecol. Clin. N. Am45(1) 45, 69–81. 10.1016/j.ogc.2017.10.003 PMC5813701 29428287

[B16] Kurtulgan H. K. Özer L. Yıldırım M. E. Ünsal E. Aktuna S. Baltacı V. (2015). Recombinant chromosome with partial 14 q trisomy due to maternal pericentric inversion. Mol. Cytogenet 8, 92. 10.1186/s13039-015-0195-7 26594242 PMC4654821

[B40] Li J. Chen Y. Wu H. Li L. (2014). Apolipoprotein E (Apo E) gene polymorphisms and recurrent pregnancy loss: a meta-analysis. J. Assist. Reprod. Genet. 31 (2), 139–148. 10.1007/s10815-013-0128-5 24221911 PMC3933605

[B17] Marshall J. D. Ludman M. D. Shea S. E. Salisbury S. R. Willi S. M. LaRoche R. G. (1997). Genealogy, natural history, and phenotype of Alström syndrome in a large Acadian kindred and three additional families. Am. J. Med. Genet. 73, 150–161. 10.1002/(sici)1096-8628(19971212)73:2<150:aid-ajmg9>3.0.co;2-y 9409865

[B18] Miryounesi M. Dianatpour M. Shadmani Z. Ghafouri-Fard S. (2016). Report of a case with trisomy 9 mosaicism. Iran. J. Med. Sci. 41 (3), 249–252. 27217611 PMC4876305

[B19] Najafi K. Mehrjoo Z. Ardalani F. Ghaderi-Sohi S. Kariminejad A. Kariminejad R. (2021). Identifying the causes of recurrent pregnancy loss in consanguineous couples using whole exome sequencing on the products of miscarriage with no chromosomal abnormalities. Sci. Rep11(1) 11, 6952. 10.1038/s41598-021-86309-9 PMC7997959 33772059

[B36] Pezzetti F. Martinelli M. Scapoli L. Carinci F. Palmieri A. Marchesini J. (2004). Maternal MTHFR variant forms increase the risk in offspring of isolated nonsyndromic cleft lip with or without cleft palate. Hum. Mutat. 24 (1), 104–105. 10.1002/humu.9257 15221800

[B20] Pinson L. Perrin A. Plouzennec C. Parent P. Metz C. Collet M. (2005). Detection of an unexpected subtelomeric 15q26.2 --> qter deletion in a little girl: Clinical and cytogenetic studies. Am. J. Med. Genet. A 138A (2), 160–165. 10.1002/ajmg.a.30939 16114049

[B21] Practice Committee of the American Society for Reproductive Medicine (2020). Definitions of infertility and recurrent pregnancy loss: A committee opinion. FertilSteril 113 (3), 533–535. 10.1016/j.fertnstert.2019.11.025 32115183

[B22] Quintero-Ronderos P. Mercier E. Fukuda M. González R. Suárez C. F. Patarroyo M. A. (2017). Novel genes and mutations in patients affected by recurrent pregnancy loss. PloS one 12 (10), e0186149. 10.1371/journal.pone.0186149 29016666 PMC5634651

[B23] Richards S. Aziz N. Bale S. Bick D. Das S. Gastier-Foster J. (2015). Standards and guidelines for the interpretation of sequence variants: A joint consensus recommendation of the American College of medical genetics and genomics and the association for molecular pathology. Med 17 (5), 405–424. 10.1038/gim.2015.30 PMC4544753 25741868

[B24] Rocha M. L. Dittmayer C. Uruha A. Korinth D. Chaoui R. Schlembach D. (2021). A novel mutation in NEB causing foetal nemaline myopathy with arthrogryposis during early gestation. Neuromusc. Disord. 31, 239–245. 10.1016/j.nmd.2020.11.014 33376055

[B25] Rudaks L. I. Nicholl J. K. Bratkovic D. Barnett C. P. (2011). Short stature due to 15q26 microdeletion involving IGF1R: Report of an additional case and review of the literature. Am. J. Med. Genet. A 155A (12), 3139–3143. 10.1002/ajmg.a.34310 22065603

[B35] Rull K. Nagirnaja L. Laan M. (2012). Genetics of recurrent miscarriage: challenges, current knowledge, future directions. Front. Genet. 3, 34. 10.3389/fgene.2012.00034 22457663 PMC3306920

[B26] Serra G. Memo L. Antona V. Corsello G. Favero V. Lago P. (2021). Jacobsen syndrome and neonatal bleeding: Report on two unrelated patients. Ital. J. Pediatr. 47 (1), 147. 10.1186/s13052-021-01108-2 34210338 PMC8252210

[B27] Singh G. Sidhu K. (2010). Bad obstetric history: A prospective study. Med. J. Armed Forces India 66 (2), 117–120. 10.1016/S0377-1237(10)80121-2 27365723 PMC4920907

[B28] Soden S. E. Saunders C. J. Willig L. K. Farrow E. G. Smith L. D. Petrikin J. E. (2014). Effectiveness of exome and genome sequencing guided by acuity of illness for diagnosis of neurodevelopmental disorders. Sci. Transl. Med. 6 (265), 265ra168. 10.1126/scitranslmed.3010076 PMC4286868 25473036

[B29] South S. T. Lee C. Lamb A. N. Higgins A. W. Kearney H. M. (2013). ACMG standards and guidelines for constitutional cytogenomic microarray analysis, including postnatal and prenatal applications: Revision 2013. Genet. Med. 15 (11), 901–909. 10.1038/gim.2013.129 24071793

[B30] Sugiura-Ogasawara M. Ozaki Y. Katano K. Suzumori N. Kitaori T. Mizutani E. (2012). Abnormal embryonic karyotype is the most frequent cause of recurrent miscarriage. Hum. Reprod. Oxf. Engl. 27 (8), 2297–2303. 10.1093/humrep/des179 22661547

[B31] Temtamy S. A. Kamel A. K. Ismail S. Helmy N. A. Aglan M. S. El Gammal M. (2007). Phenotypic and cytogenetic spectrum of 9p trisomy. Genet. Couns. 18, 29–48. 17515299

[B37] Tollånes M. C. Aarsand A. K. Sandberg S. (2011). Excess risk of adverse pregnancy outcomes in women with porphyria: a population-based cohort study. J. Inherit. Metab. Dis. 34 (1), 217–223. 10.1007/s10545-010-9231-2 20978938 PMC3026662

[B32] Torres M. R. Carrasco P. Santos C. Bueno G. Martínez-Bonet E. Carreto P. (2021). Contingent prenatal screening for frequent aneuploidies with cell-free fetal DNA analysis. Taiwan J. Obstet. Gynecol. 60 (4), 745–751. 10.1016/j.tjog.2021.05.028 34247818

[B33] Verma R. S. Rodriguez J. Dosik H. (1982). The clinical significance of pericentric inversion of the human Y chromosome: A rare "third" type of heteromorphism. J. Hered. 73 (3), 236–238. 10.1093/oxfordjournals.jhered.a109627 6212612

[B34] Xiang H. Wang C. Pan H. Hu Q. Wang R. Xu Z. (2021). Exome-sequencing identifies novel genes associated with recurrent pregnancy loss in a Chinese cohort. Front. Genet12 12, 746082. 10.3389/fgene.2021.746082 PMC8674582 34925444

[B39] Zscheppang K. Liu W. Volpe M. V. Nielsen H. C. Dammann C. E. (2007). ErbB4 regulates fetal surfactant phospholipid synthesis in primary fetal rat type II cells. American journal of physiology. Lung Cell. Mol. Physiol. 293 (2), L429–L435. 10.1152/ajplung.00451.2006 17545485

