# Supplementary material for: Identification of genetic alterations in couples and their products of conceptions from recurrent pregnancy loss in North Indian population
Source: Front Genet. 2023 May 16;14:1155211. doi: 10.3389/fgene.2023.1155211 (PMC10227573; doi:10.3389/fgene.2023.1155211)
Supplement: Supplementary file 1 [file DataSheet1.PDF]

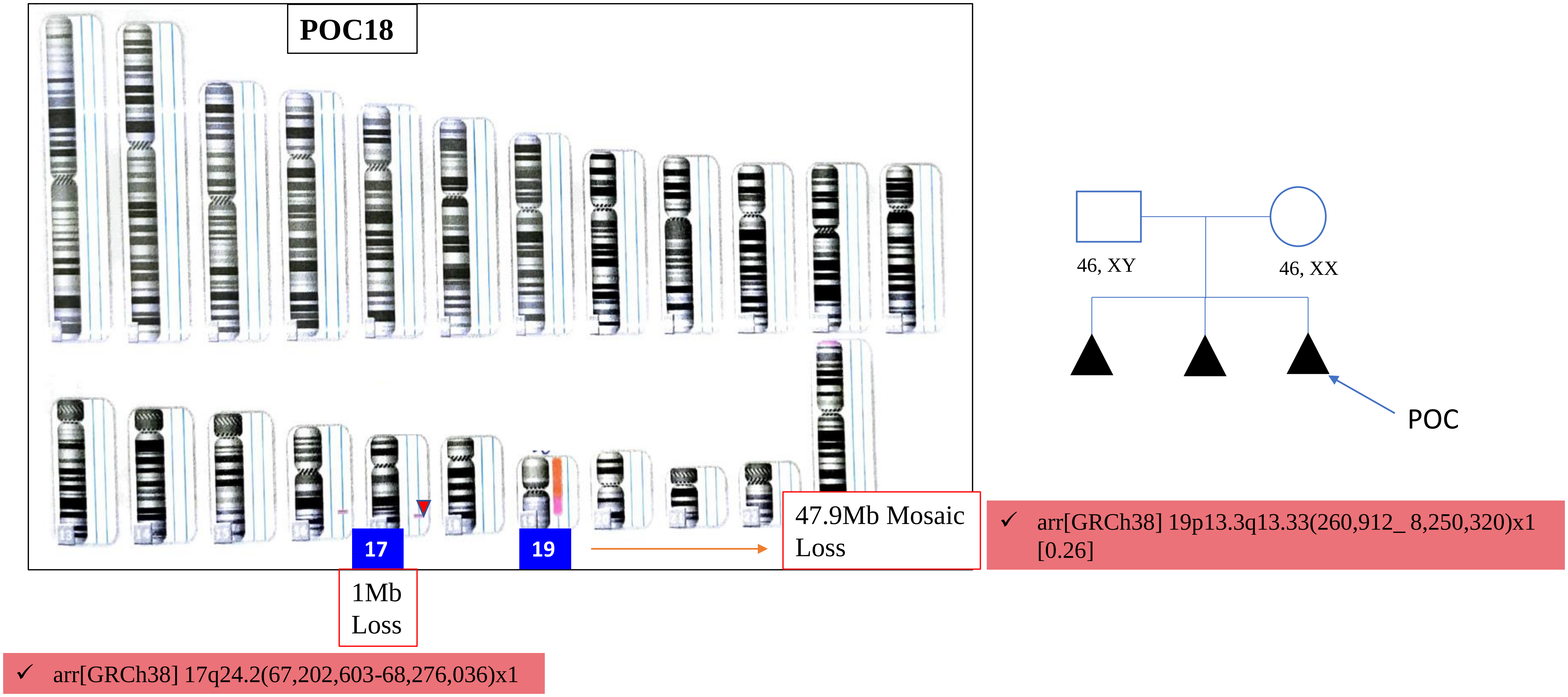

Supplementary Figure 1A: CMA picture of POC18 showing mosaic gain on chr19p13.3 and loss on 17q24.2

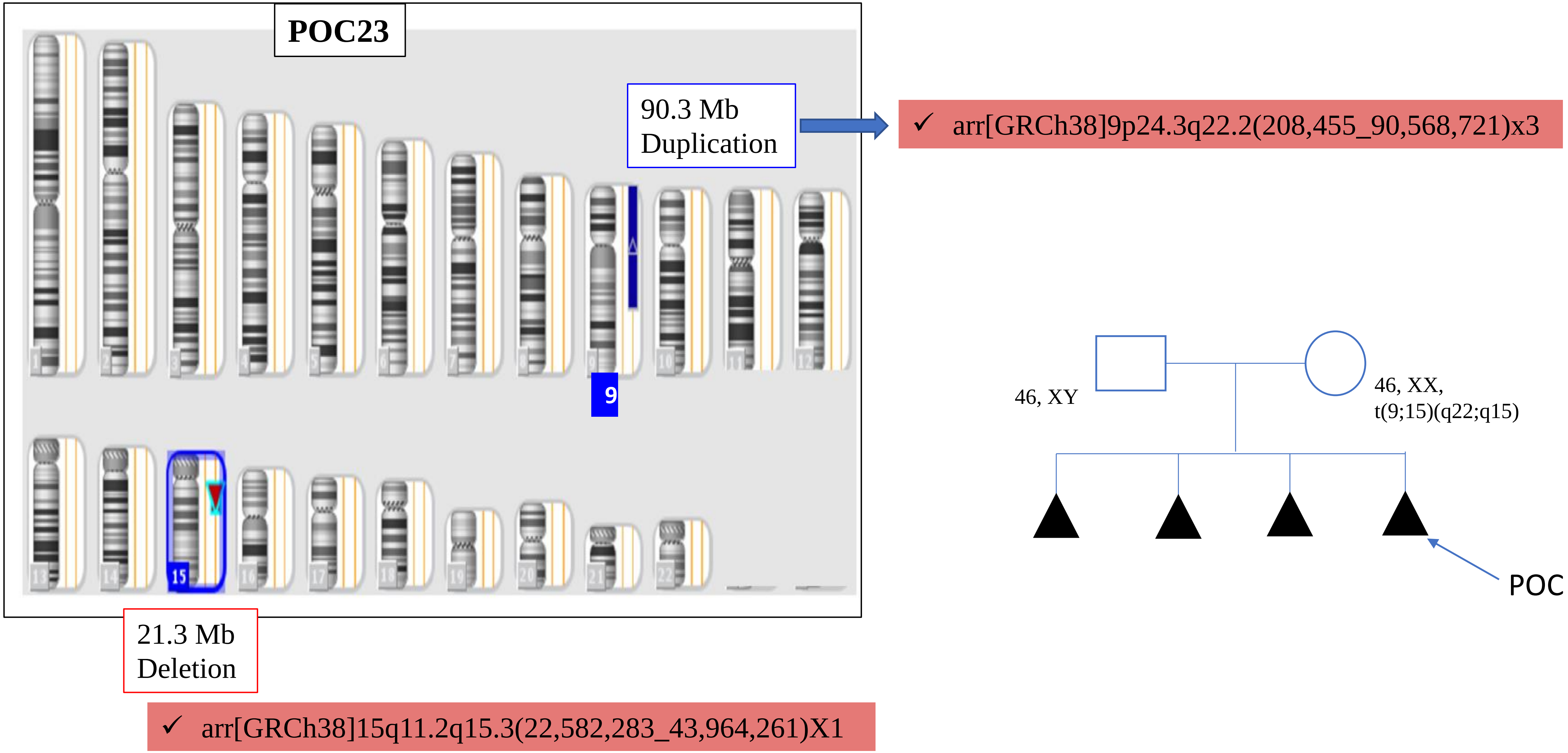

Supplementary Figure 1B: CMA picture of POC23 showing gain on chr9p24.3 and loss on 15q11.2

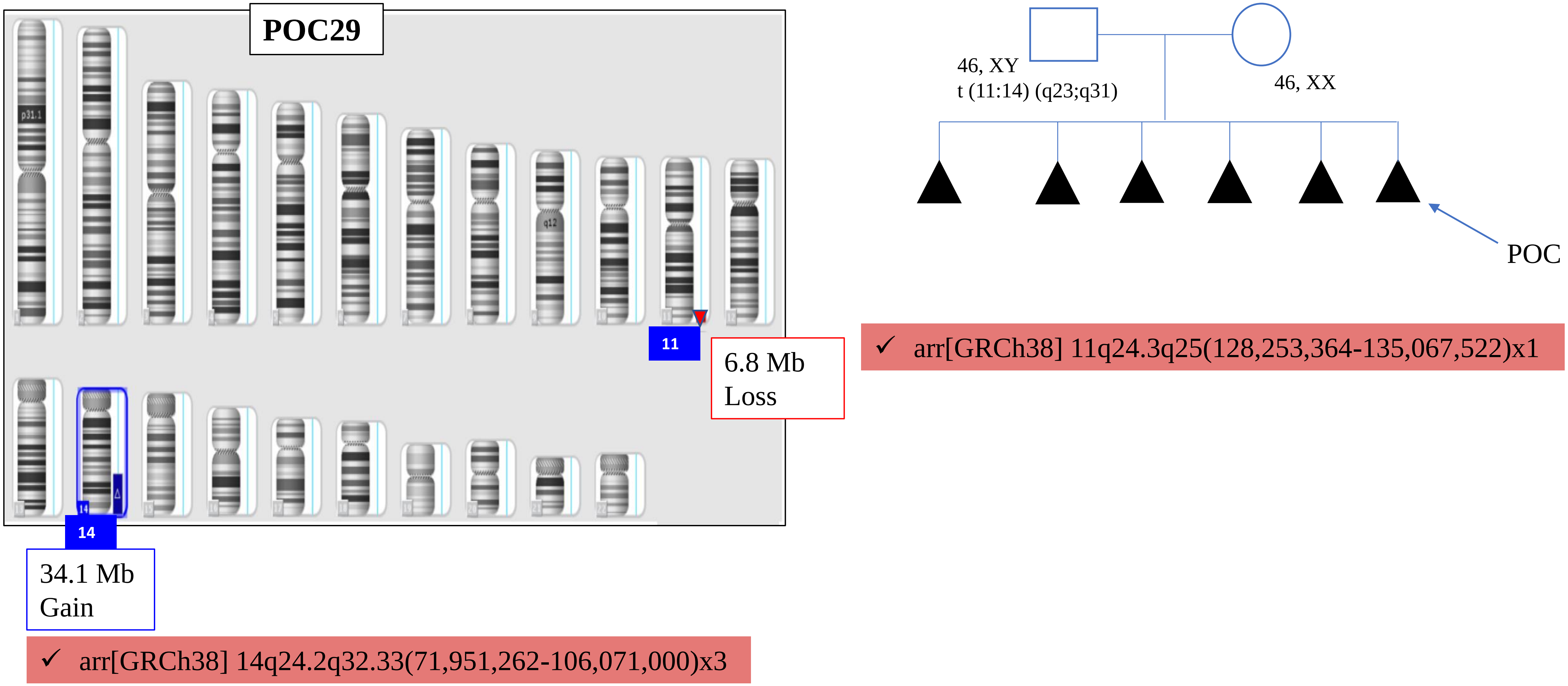

Supplementary Figure 1C: CMA picture of POC29 showing gain on chr14q24.2 and loss on 11q24.3

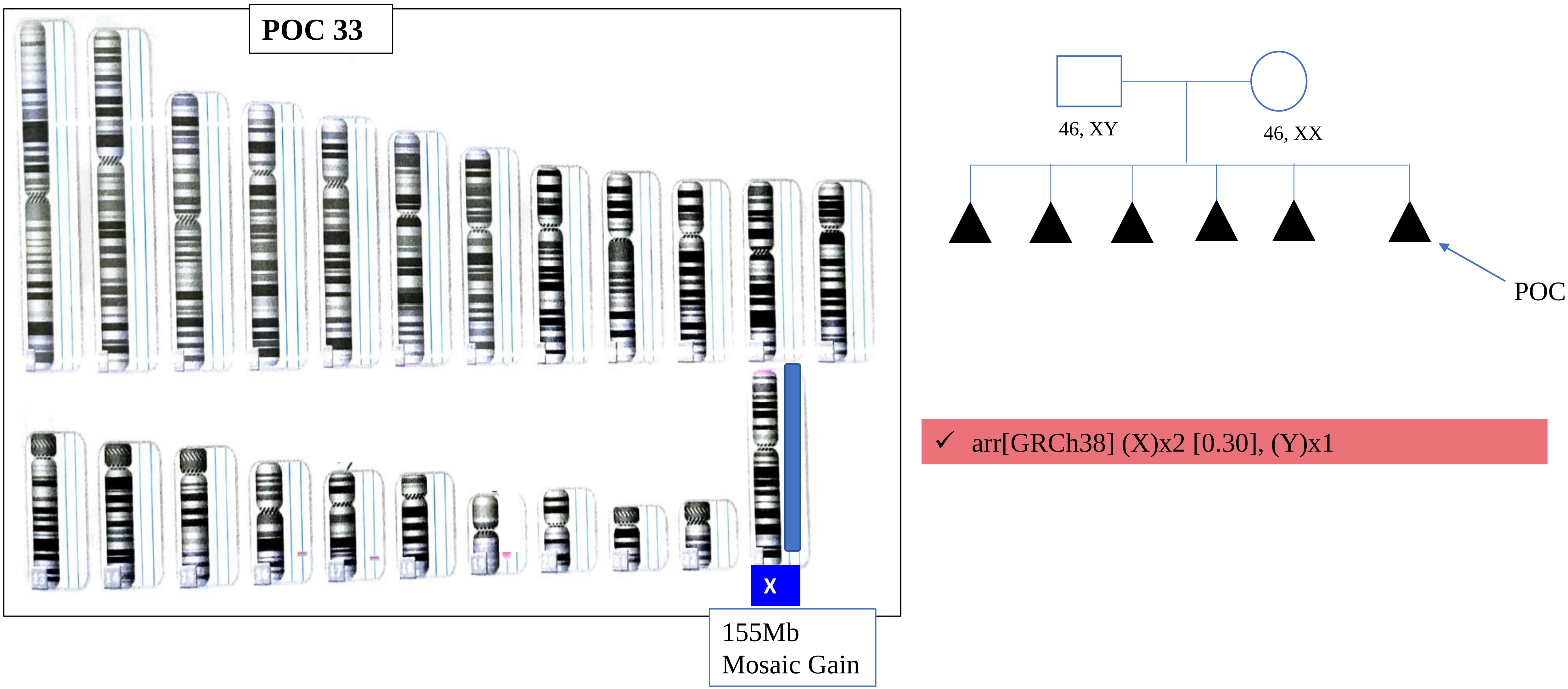

Supplementary Figure 1D: CMA picture of POC33 showing mosaic gain on chrX

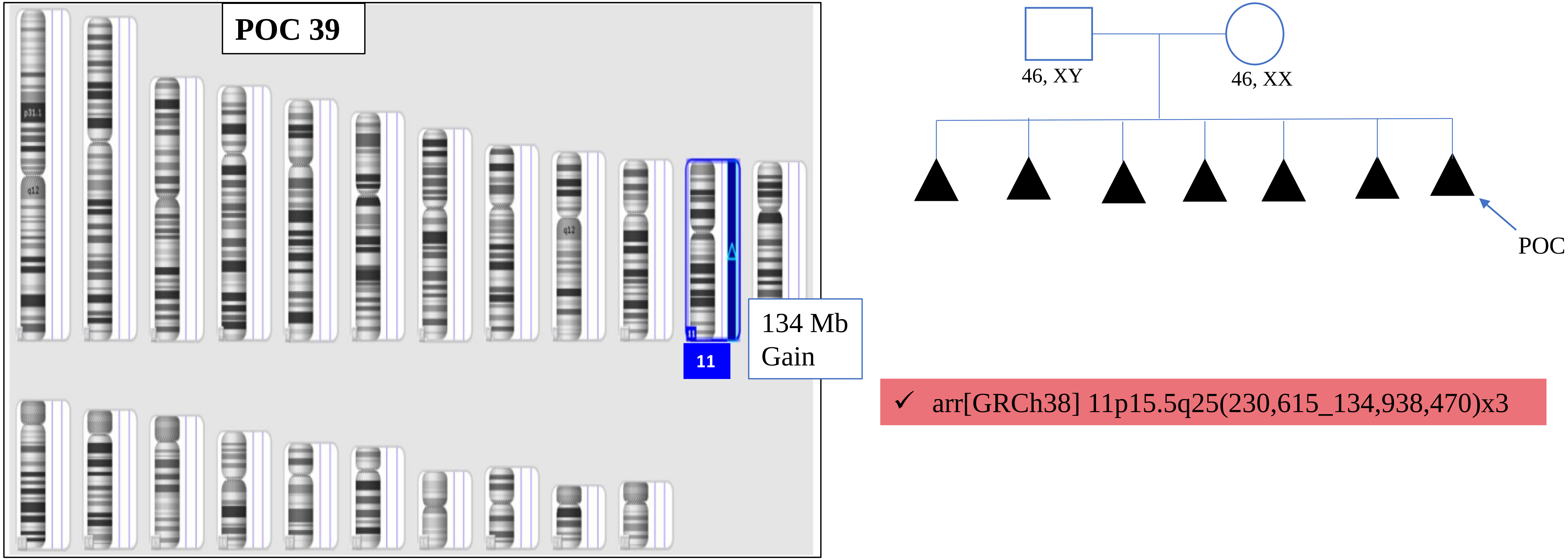

Supplementary Figure 1E: CMA picture of POC39 showing trisomy of chromosome 11

RPL50

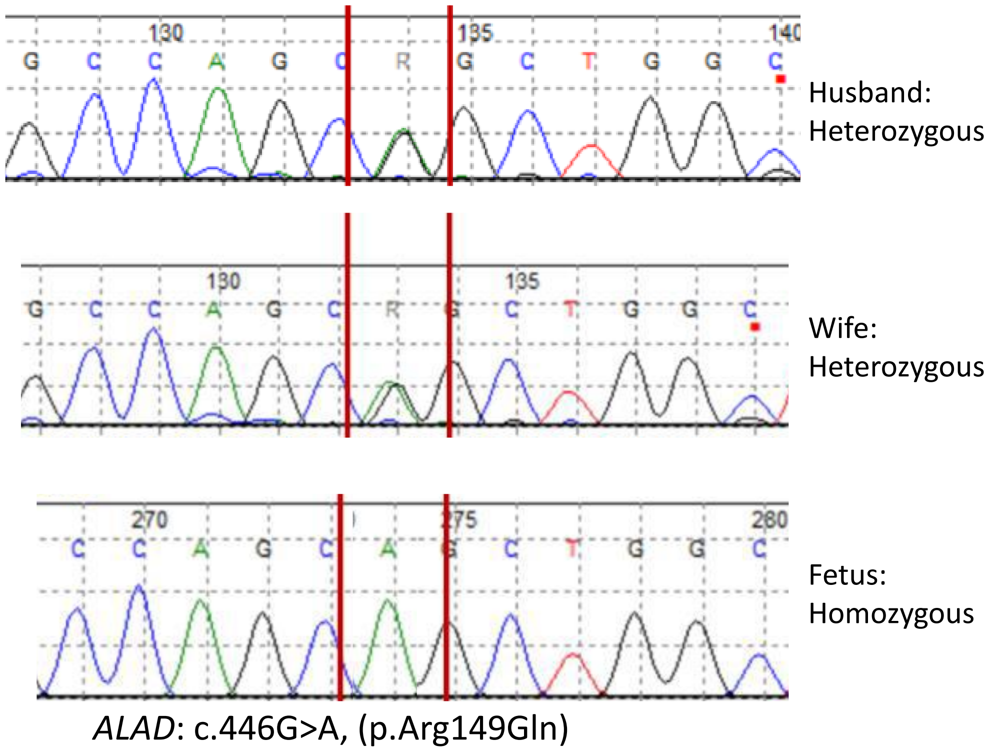

Supplementary Figure 2A: Sanger validation of Trio exome sequencing of RPL50 showing variant in *ALAD*: c.446G>A in couple (heterozygous) and fetus (Homozygous)

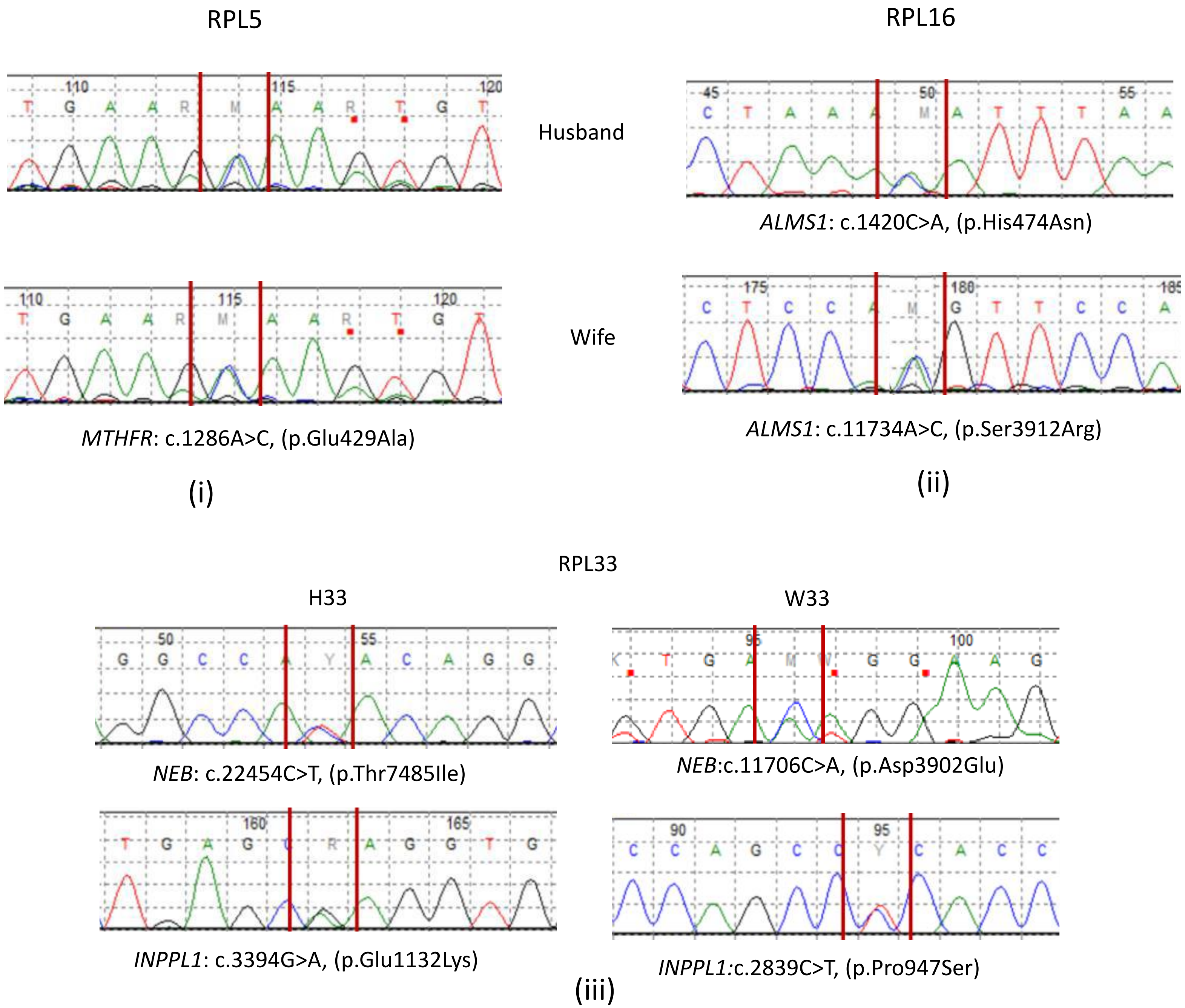

Supplementary Figure 2B: Sanger validation of couple exome sequencing

(i) RPL5 couple showing heterozygous variant in MTHFR gene

(ii) RPL16 couple showing heterozygous variants in ALMS1 gene

(iii) RPL33 couple showing heterozygous variants in NEB and INPPL1 gene
